# Accurate Memory Kernel Extraction from Discretized Time-Series Data

**DOI:** 10.1021/acs.jctc.3c01289

**Published:** 2024-04-11

**Authors:** Lucas Tepper, Benjamin Dalton, Roland R. Netz

**Affiliations:** Department of Physics, Freie Universität Berlin, 14195 Berlin, Germany

## Abstract

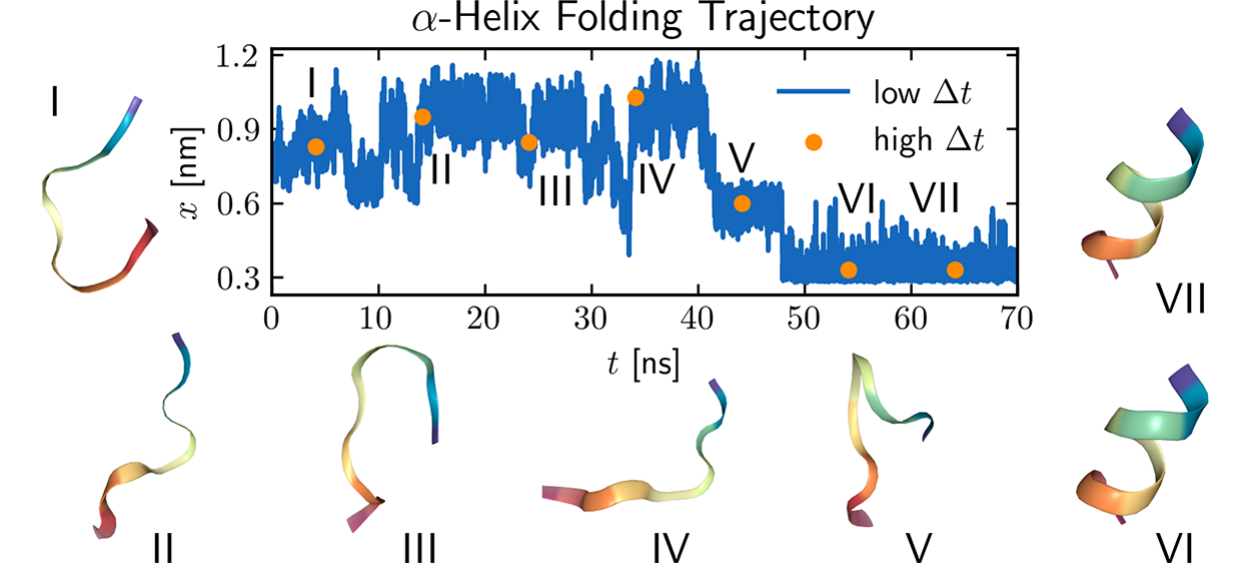

Memory effects emerge as a fundamental consequence of dimensionality reduction when low-dimensional observables are used to describe the dynamics of complex many-body systems. In the context of molecular dynamics (MD) data analysis, accounting for memory effects using the framework of the generalized Langevin equation (GLE) has proven efficient, accurate, and insightful, particularly when working with high-resolution time series data. However, in experimental systems, high-resolution data are often unavailable, raising questions about the impact of the data resolution on the estimated GLE parameters. This study demonstrates that direct memory extraction from time series data remains accurate when the discretization time is below the memory time. To obtain memory functions reliably, even when the discretization time exceeds the memory time, we introduce a Gaussian Process Optimization (GPO) scheme. This scheme minimizes the deviation of discretized two-point correlation functions between time series data and GLE simulations and is able to estimate accurate memory kernels as long as the discretization time stays below the longest time scale in the data, typically the barrier crossing time.

## Introduction

A fundamental challenge in natural sciences involves the creation of a simplified, yet accurate, representation of complex system dynamics using a low-dimensional coordinate. For instance, in spectroscopy, atomic motions are investigated solely through the polarization induced by an electromagnetic field, resulting in spectra.^1^ In the case of molecules in fluids, the myriad of interactions with the solvent is often reduced to a one-dimensional diffusion process.^2,3^ In numerous studies,^4−8^ the folding of a protein is described by a one-dimensional reaction coordinate. These diverse fields all share the common approach of projecting the complete many-body dynamics of 6N atomic positions and momenta onto a few or even a single reaction coordinate. Starting from the deterministic kinetics of a Hamiltonian system, the projection procedure yields a stochastic description based on the generalized Langevin equation (GLE),^9−11^ which, in the case of a one-dimensional coordinate *x*(*t*) and its corresponding velocity *v*(*t*), reads
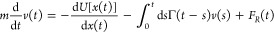
1where *m* is the effective mass of the coordinate *x*. The potential of mean force *U*(*x*) is directly available from the equilibrium probability distribution ρ(*x*) via *U*(*x*) = −*k*_B_*T* ln ρ(*x*), where *k*_B_ is the Boltzmann constant, and *T* is the absolute temperature. Non-Markovian effects arise as a direct consequence of the dimensionality reduction.^12^ In the GLE, the memory kernel Γ(*t*) weights the effect of past velocities on the current acceleration. Stochastic effects, represented by the random force *F*_*R*_(*t*), are linked to the memory function via the fluctuation–dissipation theorem in equilibrium, ⟨*F*_*R*_(0)*F*_*R*_(*t*)⟩ = *k*_B_*T*Γ(*t*). When the relaxation of the environment governing Γ(*t*) is sufficiently fast, Γ(*t*) approaches a delta kernel, and the Langevin equation emerges from the GLE. Considerable efforts have been dedicated to identifying suitable reaction coordinates to minimize memory effects and enable a Markovian description of protein folding.^4−6,8,12^

In recent works, the memory function Γ(*t*) was extracted from time series data of proteins of biological relevance, allowing the non-Markovian description of a protein’s folding kinetics in a nonlinear folding landscape. Memory effects were found to be highly relevant, both in model systems^13^ and real proteins.^14,15^ Multiple methods exist to extract memory functions from MD data. A much-used method is based on Volterra equations, which are deterministic, integro-differential equations that derive from the GLE and allow for the extraction of the memory kernel from time correlation functions.^16−19^ While Volterra equations offer good accuracy when high-quality time-series data are available, it is unclear if they remain efficient when the observations of the system are sampled with long discretization times. A recent research endeavor used an iterative scheme to approximate the memory kernel by adapting a trial kernel with a heuristic update based on the velocity autocorrelation function.^20^ Another work parametrized memory kernels by fitting correlation functions to an analytical solution of the GLE.^21^ In order to include the short and long time scales of the system dynamics, the fit included both the two-point correlation and its running integral. Both methods share the limitation of not being applicable to a nonlinear potential energy function *U*(*x*). A recent paper not suffering from such a limitation used a maximum-likelihood model to estimate the GLE parameters that best fit the given MD data.^22^ In a different work on polymer solutions, star polymers were coarse-grained to single beads interacting via a nonlinear *U*(*x*). A GLE system was set up to mimic the star polymers’ kinetics. The simulation parameters of the GLE system were iteratively changed using Gaussian Process Optimization (GPO) such that the coarse-grained and MD velocity autocorrelations were most similar.^23^ The same idea was used to estimate a joint memory kernel over multiple temperatures.^24^

Here, we consider the effects of temporal discretization, motivated by the fact that data are always discretized. For MD simulations, archived data often only contains the atomic positions at time intervals of hundreds of picoseconds to nanoseconds, as in the case of the data from the Anton supercomputer.^25^ When considering experimental data, measurement devices limit the time step of the observations, typically at the microsecond scale.^26,27^ In a prior publication, discretization effects were examined within the framework of data-driven GLE analysis. The GLE, without a potential, was solved analytically. To deal with discretization effects, the discretized mean-squared displacement and velocity autocorrelation functions were computed, allowing for the direct fitting of the memory kernel.^28^ The present work investigates how a GLE with a nonharmonic potential can be parametrized given discretized data by considering a highly nonlinear molecular dynamics test system. The Volterra-based approach is shown to be remarkably resilient to time discretizations. Where the Volterra approach ceases to function, we demonstrate that Gaussian Process Optimization is a suitable method to obtain memory kernels from discrete time series data. In matching correlation functions computed from subsampled data, we present a method to deal with the discretization effects and extend the GLE analysis to nonlinear data at higher discretizations. The choice of correlation functions involves some flexibility, demonstrating the broad applicability of our approach. For a small alanine homopeptide used as a test system, the Volterra method is suitable for discretization times that reach the memory time of about 1 ns. In comparison, the GPO method extends the range to discretization times up to the folding time of 58 ns.

## Results and Discussion

**Figure 1:**
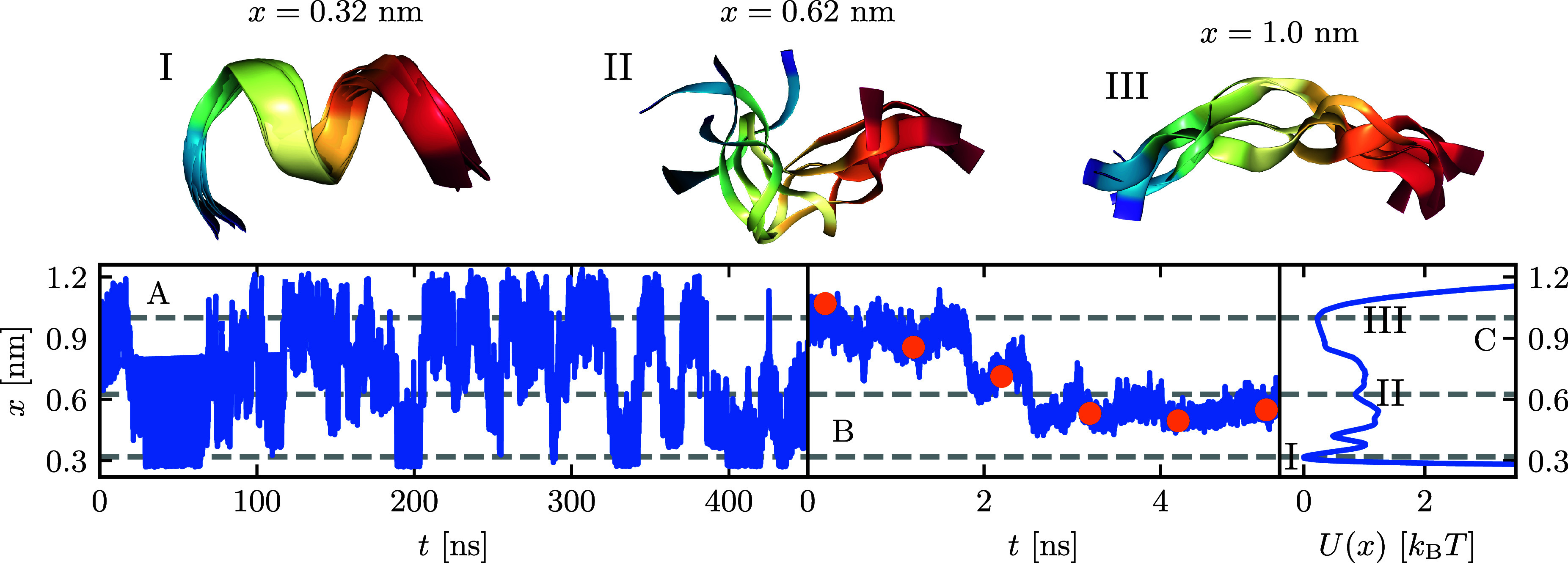
**I**-**III** Representative snapshots for different values of the mean hydrogen-bond distance reaction coordinate of Ala_9_, *x*, defined in eq 2. **A** Multiple folding and unfolding events occur within a 450 ns trajectory segment. **B** A single folding event. The orange circles indicate the time series discretized at Δ*t* = 1 ns. **C** The potential landscape *U*(*x*) for Ala_9_, computed from the trajectory at full resolution. The folded state (**I**) forms a sharp minimum at *x* = 0.32 nm. A local minimum is found at *x* = 0.62 nm (**II**). The unfolded state forms a broad minimum around *x* = 1.0 nm (**III**).

We investigate the effect of data discretization starting from a 10-μs-long MD trajectory of alanine nonapeptide (Ala_9_) in water, which was established as a sensitive test system for non-Markovian effects in our previous work.^14^ As in our original analysis, the formation of the α-helix in Ala_9_ is measured by the mean distance between the H-bond acceptor oxygen of residue *n* and the donor nitrogen of residue *n* + 4
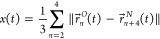
2In the α-helical state, *x* has a value of approximately 0.3 nm, the mean H-bond length between nitrogen and oxygen. The potential of mean force *U*(*x*) in Figure 1C displays several metastable states along the folding landscape; Ala_9_, therefore, is a suitable and nontrivial test system for numerical methods. Figure 1A shows a 450 ns long trajectory. To test how time discretization affects memory extraction, frames of the trajectory are left out to achieve an effective discretization time step Δ*t*. Such a discretized trajectory (orange data points for Δ*t* = 1 ns) is compared in Figure 1B to the time series at full resolution. The potential *U*(*x*) is always estimated from a histogram of the entire data set to separate time discretization from effects arising due to the undersampling of the potential (see section I in the Supporting Information).

### Volterra Equations

To extract memory kernels from time-series data, the GLE in eq 1 is multiplied by *v*(0) and averaged over time. By using the relation ⟨*F*_*R*_(*t*)*v*(0)⟩ = 0,^9,10^ one obtains the Volterra equation^14,19^
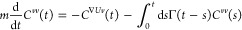
3where *C*^*vv*^(*t*) is the velocity autocorrelation function, and *C*^∇*Uv*^(*t*) is the correlation between the gradient of the potential and the velocity. By integrating eq 3 from 0 to *t*, we derive a Volterra equation involving the running integral over the kernel *G*(*t*) = ∫_0_^*t*^d*s*Γ(*s*) and insert *mC*^*vv*^(0) = *C*^∇*Ux*^(0)^14^ to obtain
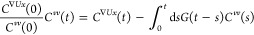
4with *C*^∇*Ux*^(*t*) being the correlation between the gradient of the potential and the position. Computing the memory kernel directly from eq 3 is possible^29,30^ but prone to instabilities.^17^ Extracting *G*(*t*) using eq 4 and computing Γ(*t*) via a numerical derivative improves the numerical stability.^17,31^ The discretization and solution of eq 4 are discussed in section II of the Supporting Information. A recent study proposed an alternative technique for extracting memory kernels by Taylor expansion of the convolution integral^32^ (we discuss the potential applicability of this Ansatz to our specific problem in section III in the Supporting Information). We fit Γ(*t*) extracted from the full-resolution data at Δ*t* = 1 fs using least-squares to a multiexponential of the form
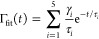
5The fitted memory times τ_*i*_ and friction coefficients γ_*i*_ are presented in Table 1. The fitting involves both Γ(*t*) and *G*(*t*), as elaborated in the Methods section, and accurately captures the MD kinetics, similar to our previous work.^14^

**Table 1:** Fitted Memory Function Parameters for Δ*t* = 1 fs According to Eq 5^a^

*i*	γ_*i*_ [u/ps]	τ_*i*_ [ps]
1	2.2 · 10^3^	0.007
2	4.4 · 10^4^	18
3	2.4 · 10^5^	370
4	6.0 · 10^4^	4100
5	4.6 · 10^3^	5700
γ_tot_ = ∑_*i*=1_^5^γ_*i*_	3.5 · 10^5^	
		1000

a The fits for Δ*t* > 1 fs are shown in section IV in the Supporting Information.

In order to estimate the impact of the non-Markovian effects on the kinetics, we turn to a heuristic formula for the mean first-passage time τ_MFP_ of a particle in a double-well potential in the presence of exponentially decaying memory.^13,33,34^ Validated by extensive simulations, the heuristic formula accurately described the non-Markovian effects occurring in the folding of various proteins.^15^ For a single exponential memory function, the heuristic formula identifies three different regimes by comparing the single memory time τ to the diffusion time scale τ_*D*_ = γ_tot_*L*^2^/*k*_B_*T*, which is the time it takes for a free Brownian particle to diffuse over a length of *L* in reaction coordinate space. The first regime is the Markovian limit, where τ ≪ τ_*D*_ and non-Markovian effects are negligible. The second regime is a non-Markovian regime where τ_*D*_/100 ≲ τ ≲ 10τ_*D*_, in which a speed-up of τ_MFP_ compared to the Markovian description is observed. The third regime occurs when τ ≳ 10τ_*D*_, where τ_MFP_ is slowed down compared to the Markovian description due to non-Markovian memory effects.

To compute τ_*D*_, we take *L* = 0.22 nm, the distance between the folded state at *x* = 0.32 nm and the barrier at *x* = 0.54 nm, the total friction γ_tot_ = ∑_*i*=1_^5^γ_*i*_ and obtain τ_*D*_ = 6.8 ns. The τ_*i*_ values in Table 1 span times from τ_1_ = 7 fs ≪ τ_*D*_ up to τ_5_ = 5.7 ns ≈ τ_*D*_. In a previous work,^15^ τ_mem_ = ∫_0_^*∞*^d*ss*Γ(*s*)/∫_0_^*∞*^d*s* Γ(*s*), the first moment of the memory kernel, was proposed as the characteristic time scale for a multiscale memory kernel. For the memory kernel in Table 1, we find τ_mem_ = 1 ns, correctly predicting the non-Markovian speed-up of τ_MFP_ that a previous study demonstrated for Ala_9_.^14^ In this work, we will establish τ_mem_ as the limit for the discretization time Δ*t*, beyond which the Volterra method ceases to produce accurate results.

**Figure 2:**
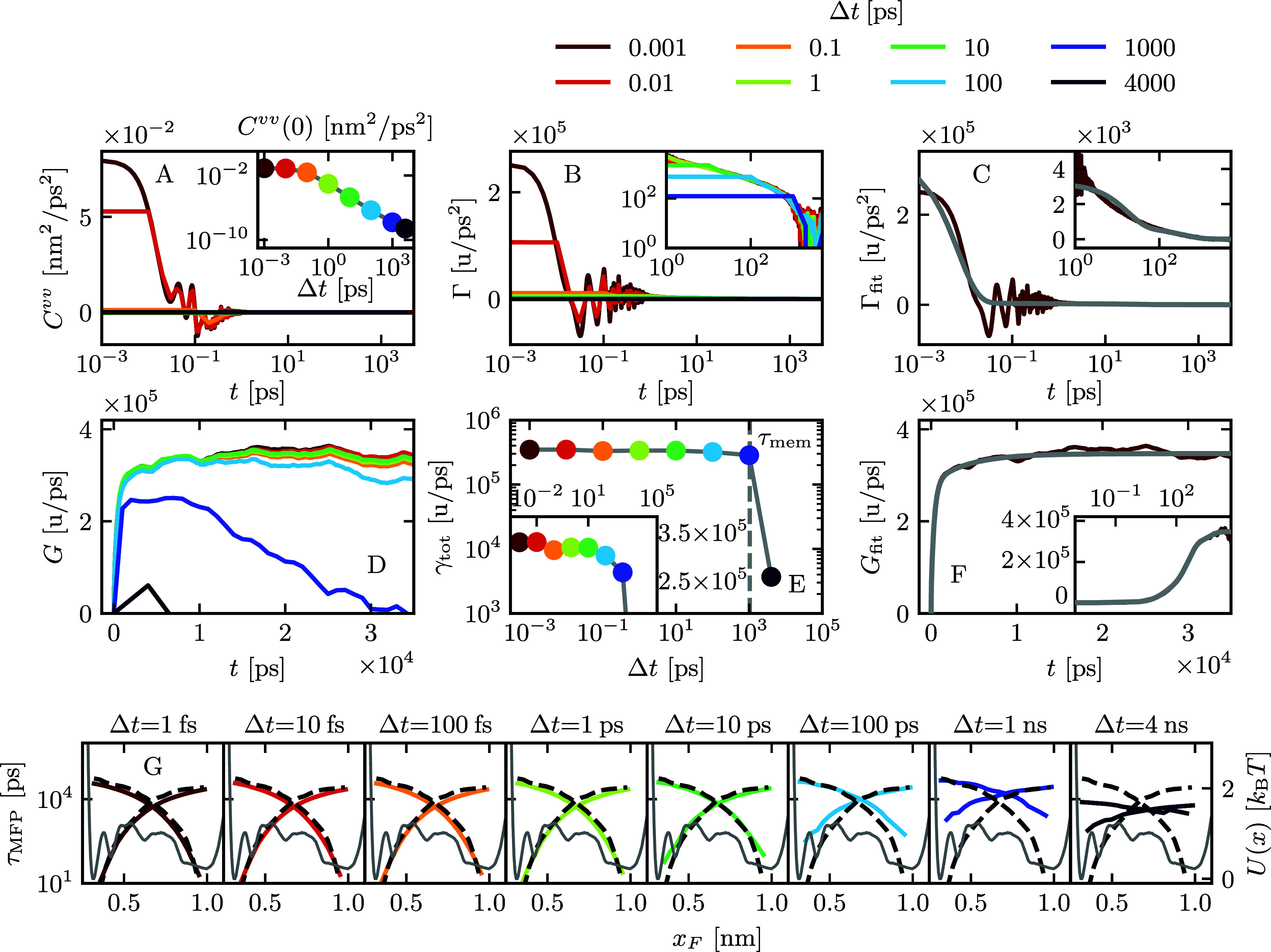
Memory extraction by the inversion of the Volterra eq 4 for different discretization times Δ*t*, using data from MD simulations of Ala_9_. **A** Velocity autocorrelation *C*^*vv*^(*t*). **B** Memory kernel Γ(*t*), from numerical differentiation of *G*(*t*). **C** Multiexponential fit of Γ(*t*) computed for Δ*t* = 1 fs (gray) compared to the corresponding numerical data (dark red). The fitted parameters are shown in Tables 1 and S1. **D** Running integral over the memory kernel *G*(*t*). **E** Total friction γ_tot_, computed from the exponential fits of the kernels. The vertical broken gray line indicates τ_mem_ = ∫_0_^*∞*^d*ss*Γ(*s*)/∫_0_^*∞*^d*s* Γ(*s*) = 1 ns. **F** Fit of *G*(*t*) (gray) computed at Δ*t* = 1 fs compared to corresponding numerical data (dark red). **G** Comparison of the mean first-passage times τ_MFP_ computed from the MD data (black broken lines) to τ_MFP_ obtained from GLE simulations using kernels extracted at different Δ*t*s (colored lines).

In the following, the full-resolution kernel obtained for a time step of Δ*t* = 1 fs will serve as a reference for results using a higher Δ*t*. Comparing the extracted *G*(*t*) with the corresponding fit according to eq 5 (red line) in Figure 2F shows no significant differences in the long time limit. Figure 2C shows oscillations of the extracted Γ(*t*) for *t* < 1 ps, which are discarded by the exponential fit. As we will show later, they do not play a role in the kinetics. For both Γ(*t*) in Figure 2B and *C*^*vv*^(*t*) in Figure 2A, the oscillations disappear for Δ*t* ≥ 0.1 ps, indicating that they are caused by subpicosecond molecular vibrations. Moreover, the value of Γ(*t*) for *t* < 1 ps is consistently attenuated as Δ*t* increases, mirroring the same trend observed in *C*^*vv*^(0), as illustrated in the inset of Figure 2A. In contrast, Γ(*t*) for *t* > 1 ps in the inset of Figure 2B shows an exponential decay that is well preserved for all Δ*t* < 1 ns. The running integral *G*(*t*) in Figure 2D stays mostly unchanged for discretizations smaller than Δ*t* < 1 ns. This demonstrates that the Volterra extraction scheme is accurate for discretization times below the mean memory time, i.e. for Δ*t* < τ_mem_ = 1 ns.

The multiexponential kernel in eq 5 allows for the efficient numerical simulation of the GLE by setting up a Langevin equation where the reaction coordinate *x* is coupled harmonically to one overdamped, auxiliary variable per exponential component^10,35^ (see section V in the Supporting Information). Utilizing this simulation technique, Figure 2G compares profiles for the mean first-passage times τ_MFP_ originating from both the folded and unfolded states. For Δ*t* ≤ 10 ps, the τ_MFP_ values obtained from the GLE simulations (colored lines) closely align with those derived from MD simulations (black broken lines), thereby manifesting the precise correspondence between the non-Markovian GLE description and the kinetics observed in the MD simulation. In Figure 2E, we present the asymptotic limit lim_*t*→*∞*_*G*(*t*), representing the total friction coefficient γ_tot_ of the system, estimated by summing the individual γ_*i*_ values obtained from the exponential fits. When Δ*t* ≥ 1 ns, we find that *G*(*t*) does not show a plateau value in the long-time limit. Consequently, this leads to a notable discrepancy between the τ_MFP_ profiles presented in Figure 2G and their MD counterparts for Δ*t* ≥ 1 ns. Combining the information provided in Figure 2D, E, and G, it becomes evident that the extracted profile of *G*(*t*), the total friction γ_tot_, and folding times τ_MFP_ all deviate significantly from the MD reference data when the discretization time approaches the memory time τ_mem_. As a result, we assert that Volterra extraction becomes inadequate when the discretization time exceeds the memory time τ_mem_. In Section VI of the Supporting Information, we demonstrate that the failure of the Volterra extraction scheme for large Δ*t* is mostly due to discretization effects in the potential gradient-position correlation function *C*^∇*Ux*^(*t*).

### Gaussian Process Optimization

So far, we have demonstrated that the Volterra equation can be used to extract a consistent memory kernel for a wide range of discretization times up to Δ*t* ≈ τ_mem_. The resulting GLE faithfully captures the underlying kinetics when judged by τ_MFP_ for discretizations below the memory time scale τ_mem_ but fails when exceeding it. Given that the discretization time may exceed the dominant memory time scale in typical experimental settings, an improved method is clearly desirable. In the following, we describe a scheme that is not based on the Volterra equation and allows the extraction of Γ(*t*) for Δ*t* that significantly exceeds τ_mem_. For this, we use a matching scheme between the discretized time correlation functions of the MD reference system *C*^MD^(*n*Δ*t*) and of the GLE *C*^GLE^(*n*Δ*t*, θ) via the mean-squared loss
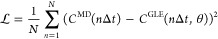
6The type of correlation function will be specified later. The loss is evaluated over *N* samples, where *N* is determined based on the decay time of the correlation (see Table S3). In an iterative optimization, the friction and memory time parameters in eq 5 that serve as the GLE parameters θ = (γ_1_, τ_1_, ..., γ_5_, τ_5_) are updated, and the GLE is integrated using a simulation time step *δt* chosen small enough that discretization effects in the GLE simulations are negligible. For the sake of comparability, we maintain a constant mass value of *m* = 31.4 u, derived using the equipartition theorem according to *m* = *k*_B_*T*/⟨*v*^2^⟩, from the MD data. In fact, the precise value of *m* has no significant influence on the method’s outcome since it can be accommodated within the kernel. Furthermore, the system’s inertial time τ_m_ = *m*/γ_tot_ = 0.09 fs is markedly shorter than all other relevant time scales, leading to an overdamped system in which the mass value is irrelevant. To find the best parameter set, θ, the choice of the optimizer is crucial. The loss , defined in eq 6, is inherently noisy due to the stochastic integration of the GLE and possesses, in general, many local minima in a high-dimensional space. Faced with such a task, common gradient-based or simplex methods fail.^36,37^ Genetic algorithms present a powerful alternative but require many sample evaluations.^38−40^ Given the computational cost of a converged GLE simulation, we choose Gaussian Process Optimization (GPO)^41−43^ as a method to minimize . GPO builds a surrogate model of the real loss  that incorporates noise^44−46^ and allows for nonlocal search^47,48^ (see section VII in the Supporting Information). As an active learning technique, it guides the sampling of new parameters, improving optimization efficiency.^49−51^

**Figure 3:**
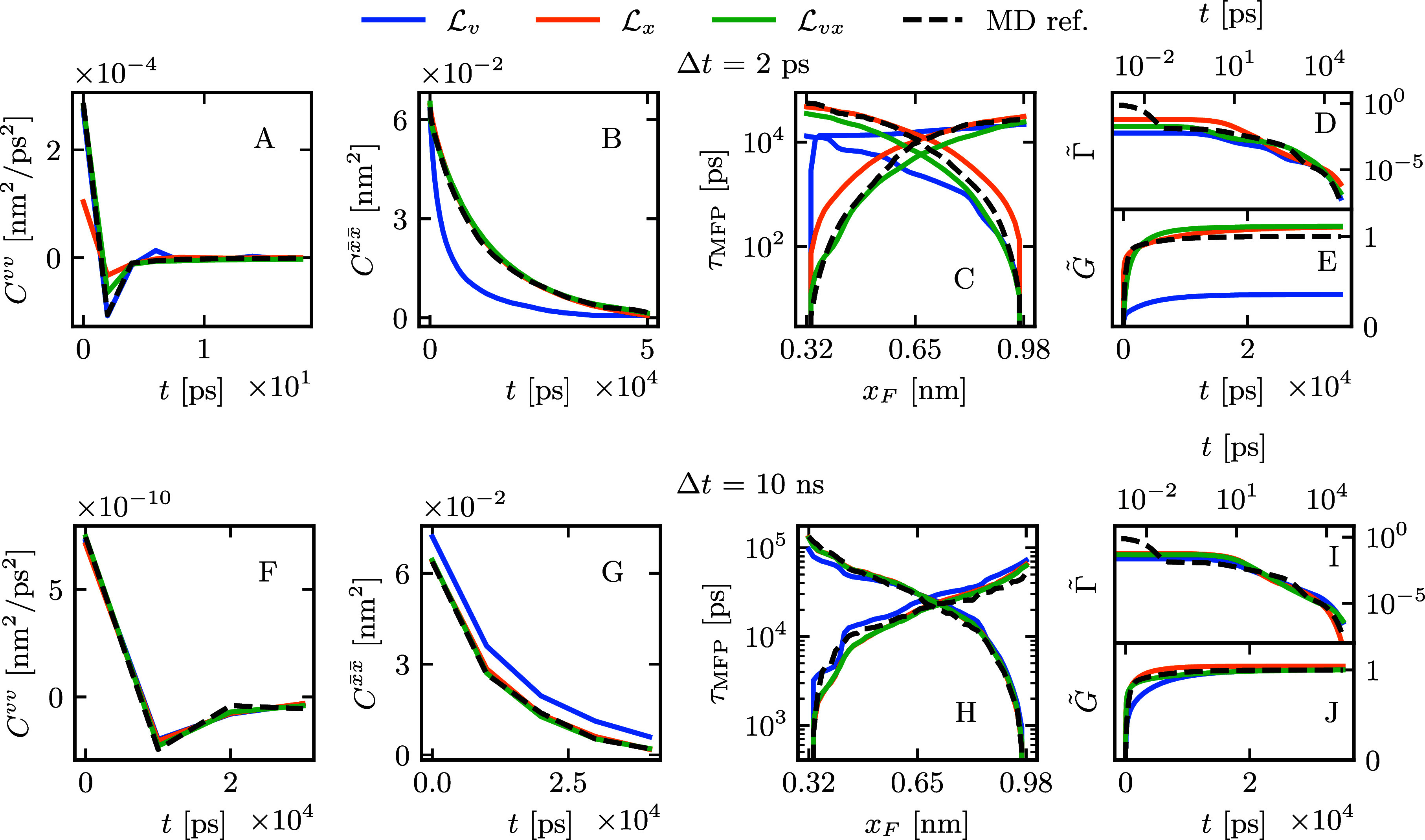
To visualize the Gaussian Process Optimization (GPO), we plot the mean of observables over the 10 best optimization runs. We compare GPO results using the loss  (blue), based on *C*^*vv*^(*t*),  (orange), based on the autocorrelation of the position *x̅*(*t*) = *x*(*t*) – ⟨*x*⟩ and , a linear combination of  and  (green). For Δ*t* = 2 ps, we compare the observables **A***C*^*vv*^(*t*), **B**, **C** τ_MFP_, **D***Γ̃*(*t*) = Γ(*t*)/Γ(0), and **E***G̃*(*t*) = *G*(*t*)/*G*(0) to the MD reference (black broken line). Equally, for Δ*t* = 10 ns, we show **F***C*^*vv*^(*t*), **G**, **H** τ_MFP_, **I***Γ̃*(*t*), and **J***G̃*(*t*). The kernels in **D**, **E**, **I**, and **J**, parametrized by eq 5, are plotted as time-continuous functions.

**Figure 4:**
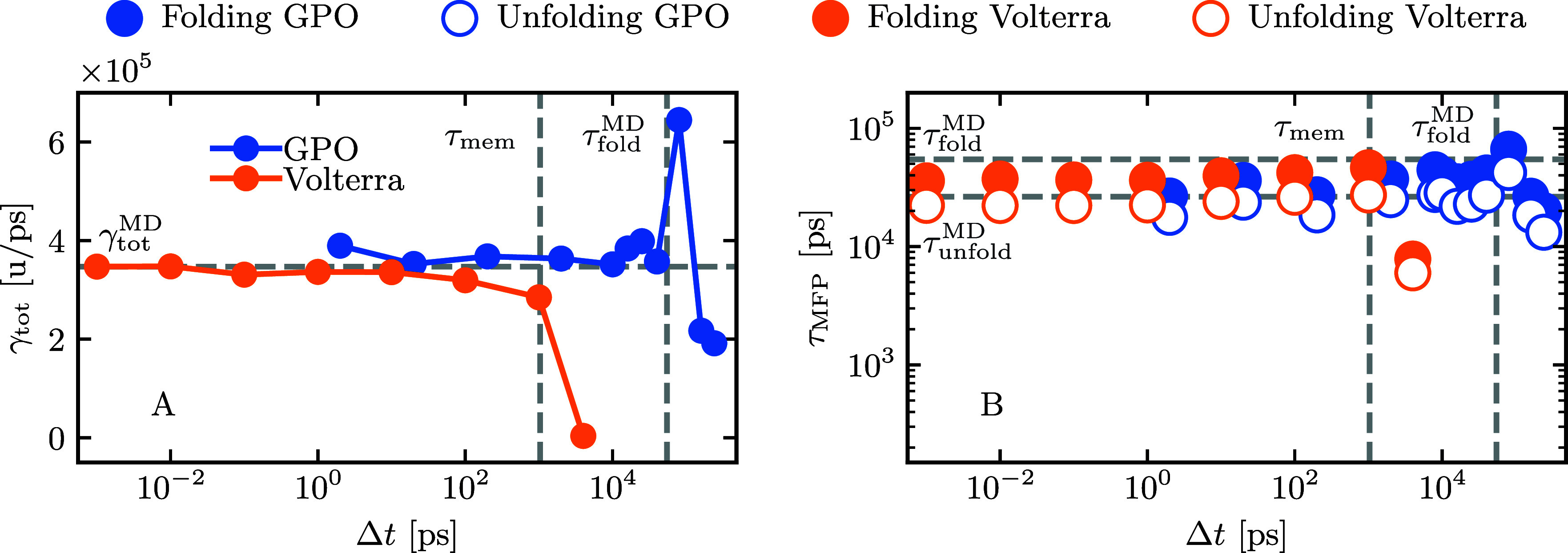
**A** The total friction γ_tot_ = ∑_*i*=1_^5^γ_*i*_ obtained via the Volterra scheme (orange) is constant for discretizations of Δ*t* < 1 ns. For Δ*t* higher than the memory time τ_mem_ = 1 ns, it decreases until the extraction fails. Gaussian process optimization (GPO, blue) estimates the correct friction for much higher Δ*t*. The horizontal gray line shows γ_tot_^MD^, the total friction extracted directly from the MD data. **B** The folding and unfolding mean first-passage times from GLE simulations with kernels extracted at different discretizations, given by the mean time it takes the system to first reach from *x* = 0.32 nm to *x* = 0.98 nm (unfolding) and reverse (folding). The MD folding times, τ_fold_^MD^ = 58 ns and τ_unfold_^MD^ = 26 ns, are indicated as horizontal gray lines τ_mem_ = 1 ns and τ_fold_^MD^ as vertical gray lines. The GPO estimates the correct folding and unfolding times up to Δ*t* ≈ τ_fold_^MD^, significantly higher than the Volterra scheme.

In principle, any correlation function can serve as an optimization target. Figure 2A shows that the velocity autocorrelation function *C*^*vv*^(*t*) decays to zero after about 1 ps, while Figure 3B,G shows that , the autocorrelation of the position *x̅*(*t*) = *x*(*t*) – ⟨*x*⟩, decays much more slowly over about 50 ns. With such a difference in the decay times of the two correlations, we define two losses based on eq 6, , using *C*^*vv*^(*t*), and , using , anticipating that the two correlations probe different scales of the dynamics. Furthermore, we define , a linear combination of  and , to test if including both correlations in the loss function improves the quality of the GLE parameters. The parameter α is selected for each Δ*t* to achieve a balanced weighting between the two losses and is tabulated in Table S3 in the Supporting Information. For every GP optimization, 300 different θ values are evaluated via 18-μs-long GLE simulations each. The 10 θ samples with the lowest loss form the basis for the following analysis. When optimizing the loss function with a discretization of Δ*t* = 2 ps, Figure 3A illustrates that  (blue) accurately replicates the MD reference for *C*^*vv*^(*t*), whereas  (orange) exhibits discrepancies. Conversely, in Figure 3B,  perfectly reproduces , while  struggles to do so. Remarkably, the combined loss function  (green) successfully aligns with both reference correlations simultaneously. To evaluate the quality of the GLE parameters, Figure 3C provides a comparison of the mean first-passage times τ_MFP_ between GLE results from the GPO solutions and the MD reference. We calculate τ_MFP_ for GPO-based GLE simulations and the MD reference using identical discretizations Δ*t*. Notably, we observe that  fails to align with the MD reference, whereas both  and  exhibit consistency with it. This outcome underscores the insufficiency of *C*^*vv*^(*t*) in capturing the slow kinetics of barrier crossing. A comparison of τ_MFP_ between  and  reveals a slightly better correspondence to the MD reference for , signifying that the inclusion of *C*^*vv*^(*t*) improves the optimization. Examining the obtained memory kernels in Figure 3D-E, all loss functions yield kernels that largely conform to the exponential fit of the MD reference but exclude the first memory component with a decay time of approximately τ_1_ ≈ 7 fs. Both  and  correctly identify the plateau of *G*(*t*), while  underestimates it, which we identify as the origin for the failure to correctly predict τ_MFP_. Next, we evaluated the performance of the GPO for discretization times exceeding τ_mem_. In Figure 3F-J, we show the results for Δ*t* = 10 ns, demonstrating that the GPO approach yields similar results for all differently defined loss functions. The discretized *C*^*vv*^(*t*), , and τ_MFP_ are in perfect agreement with the MD reference. The kernels agree for all but the lowest times. To confirm that the increased discretization used for the τ_MFP_ computation does not introduce any bias into the results, we perform an additional comparison of τ_MFP_ computed at the full-time resolution of Δ*t* = 2 fs (see Figure S4 in the Supporting Information). Figure 4 provides a comparison of the performance of the Volterra and GPO approaches across various discretizations. This comparison focuses on the overall friction, folding, and unfolding mean first-passage times, as these observables are not included in the GPO optimization process. As shown in the previous section, the applicability of the Volterra method is limited to discretizations below memory time τ_mem_ = 1 ns. Extraordinarily, the GPO approach can surpass the boundary set by the memory time and estimates folding times with good accuracy for discretizations up to Δ*t* = 40 ns. This limit roughly corresponds to the mean time it takes the system to fold, τ_fold_^MD^ = 58 ns, which is given by the mean first-passage time from the unfolded state at *x* = 0.98 nm to the folded state at *x* = 0.32 nm. For the highest discretization time tested, Δ*t* = 240 ns, the GP optimization still finds meaningful folding times, while underestimating the total friction.

## Conclusions

We investigate the effect that time discretization of the input data has on memory extraction. As a specific example, we consider MD time-series data of the polypeptide Ala_9_. Computing a memory kernel via the inversion of the Volterra eq 4 requires the velocity autocorrelation and potential gradient-position correlation function. These autocorrelations change significantly as a result of increasing time discretization, and with it a surrogate kernel is obtained that differs from the full-resolution kernel. Our key finding is that given a discretization time lower than the characteristic memory time, the Volterra approach can compute a kernel that reproduces the kinetics of the MD system. Here, we define the characteristic memory time τ_mem_ via the first moment of the memory kernel, taking into account all decay times of the kernel, and find τ_mem_ = 1 ns for Ala_9_. By extracting the memory kernel from MD trajectories at different discretizations, we show that the Volterra approach is able to reproduce the kinetics when the discretization time Δ*t* is below τ_mem_.

To also cover the important regime when Δ*t* > τ_mem_, we introduce a Gaussian Process Optimization (GPO) scheme based on matching discretized time correlation functions of the reference and the GLE system. We test losses based on the velocity and position autocorrelation functions, for which GPO yields memory kernels very similar to the Volterra scheme and is able to reproduce the reaction-coordinate dynamics and the folding times.

We demonstrate the effectiveness of GPO for discretization times up to the folding time of τ_fold_^MD^ = 58 ns, about 50 times higher than the highest discretization for which the Volterra approach is applicable. As elaborated in previous works,^13−15^ memory can affect the kinetics of protein barrier crossing on time scales far exceeding the memory time, up to the longest time scale of the system. Therefore, the presented GPO approach is expected to extend the applicability of non-Markovian analysis to a wide range of discretized systems not suitable for the Volterra method.

In fact, the GPO analysis is not limited to data from MD simulations but can be used whenever encountering highly discrete experimental data. The application to data from single-molecule experiments^52−54^ is a promising venue for future research.

## Methods

The MD simulation data is taken from our previous publication, see^14^ for details. The MD simulation has a simulation time step of *δt* = 1 fs, while all GLE simulations use a time step of *δt* = 2 fs. In the computation of the hb4 coordinate (eq 2), the distances are computed between the oxygens of Ala2, Ala3, and Ala4 and the nitrogens of Ala6, Ala7, and Ala8, where Ala1 is the alanine residue at the N-terminus of the polypeptide of Ala_9_.

All analysis code is written in Python^55^ or Rust.^56^Table S3 shows the weights α for the loss , which includes *C*^*vv*^(*t*) and . The memory kernels are fitted using the differential evolution algorithm implemented in the Python package ’scipy’^57^ by minimizing a mean-squared loss, including both the kernel and the running integral over the kernel, , where  is the mean-squared loss of the kernel and  is the mean-squared loss of the running integral of the kernel. The resulting kernels and values for α_mem_ are shown in Table S1.

The GPO is performed using the ‘GaussianProcessRegressor’ implemented in the Python package ‘scikit-learn’,^58^ using 10 optimizer restarts. When computing the loss , the correlation functions are evaluated over a finite number of sample points, *N*, always beginning with *t* = 0. The number of sample points *N* is given in Table S3. To minimize the expected improvement in eq S16 or maximize the standard deviation in eq S17, we use the ‘L-BFGS-B’ method implemented in ‘scipy’,^57^ starting from 200 random samples drawn uniformly over the space of the parameters θ (see Table S2). When performing the analysis of the GPO on the basis of the 10 best runs, the integrations are repeated with a different seed for the random number generator used in the GLE integration, ensuring that the observables are reproduced by different integration runs with the same GLE parameters θ.

## Supporting Information Available

Additional derivations, details for numerical implementations, detailed numerical results, and additional figures (PDF)
