# Supplementary material for: Accurate Memory Kernel Extraction from Discretized Time-Series Data
Source: J Chem Theory Comput. 2024 Apr 11;20(8):3061–8. doi: 10.1021/acs.jctc.3c01289 (PMC11044577; doi:10.1021/acs.jctc.3c01289)
Supplement: Supplementary file 1 — ct3c01289_si_001.pdf [file ct3c01289_si_001.pdf]

Supporting Information: Accurate Memory Kernel Extraction From Discretized Time-Series Data

Lucas Tepper, Benjamin Dalton, and Roland R. Netz*

Department of Physics, Freie Universität Berlin, 14195 Berlin, Germany

E-mail: rnetz@physik.fu-berlin.de

I Subsampling of the Potential

For the computation of the correlation between the gradient of the potential and the position $C^{\nabla Ux}(t)$, we estimate the density $\rho(x)$ from the trajectory via a histogram and compute the potential using $U(x) = -k_B T \ln \rho(x)$. Fig S1 shows how the estimated $U(x)$ would behave if frames were left out to achieve an effective time step Δt. In the main text and all further sections, the potential $U(x)$ is always estimated from a histogram of the entire data. Section VIII explains how to compute $C^{\nabla Ux}(t)$ from $U(x)$.

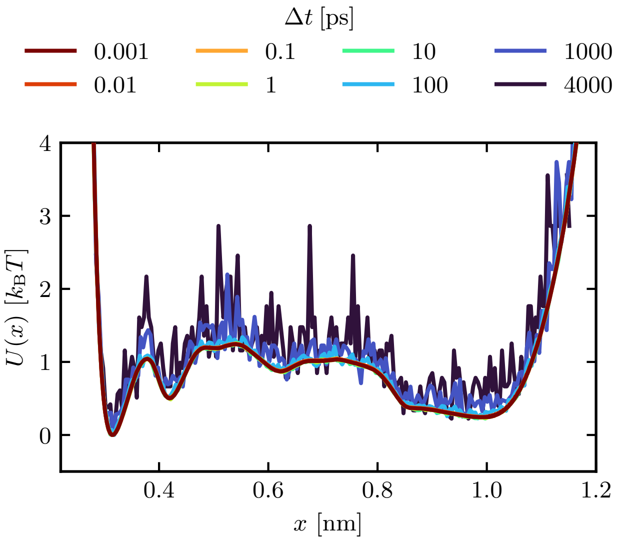

Figure S1: When generating a trajectory at an effective time step Δt from the 10 μs trajectory of Ala_9, the estimated potential visibly changes for $\Delta t \geq 100$ ps. We show the potential for all Δt used in the main text. All memory extractions in the main text use the potential at full resolution, i.e. $\Delta t = 1$ fs.

II Discretization of the Volterra Equation

Starting from Eq. 4, we discretize all functions of time and use the trapezoidal rule for the integral, obtaining[S1]

$$G_n = \frac{2}{\Delta t C_0^{vv}} \left(C_n^{\nabla Ux} - \frac{C_0^{\nabla Ux}}{C_0^{vv}} C_n^{vv} - \Delta t \sum_{i=1}^{n-1} G_{n-i} C_i^{vv} \right). \tag{S1}$$

Regarding the computation of $C^{vv}(t)$, Section IX tests different numerical differentiation schemes to compute the velocities $v(t)$ from the positions $x(t)$.

III Comparison to the Integrative GME Method

A recent paper[S2] presents the Integrative GME Method, offering a non-Markovian extension to Markov state models based on the generalized master equation (GME). It surpasses the accuracy of previous GME-based methods by introducing a Taylor expansion of the convolution integral involving the memory kernel $\Gamma(t)$ and the time-dependent transition probability $T(t)$ (see[S2] for more details on the transition probability). In brief, the central step is to expand the kernel integral as

$$\begin{aligned}\int_0^t \mathrm{d}s T(t-s)\Gamma(s) &= \int_0^t \mathrm{d}s \left[T(t) + \sum_{n=1}^{\infty} \frac{1}{n!} \frac{\mathrm{d}^n T(t)}{\mathrm{d}t^n} (-s)^n\right] \Gamma(s), \\ &= T(t) M_0(t) + \sum_{n=1}^{\infty} \frac{(-1)^n}{n!} \frac{\mathrm{d}^n T(t)}{\mathrm{d}t^n} M_n(t),\end{aligned} \tag{S2}$$

where we have defined the kernel moments as $M_n(t) = \int_0^t \mathrm{d}s \Gamma(s) s^n$. Now, if t in equation S2 is larger than the time τ_k it takes $\Gamma(t)$ to decay to zero, the moments $M_n(t)$ become constant $M_n(t > \tau_k) = M_n(\tau_k) := M_n$. With this, equation S2 becomes

$$\int_0^t \mathrm{d}s T(t-s)\Gamma(s) = T(t) M_0 + \sum_{n=1}^{\infty} \frac{(-1)^n}{n!} \frac{\mathrm{d}^n T(t)}{\mathrm{d}t^n} M_n. \tag{S3}$$

Equation S3 can be used to iteratively solve for the transition probability $T(t)$ without directly computing the memory terms M_n.[S2] To apply equation S3 to our task, we replace $T(t)$ by $C^{vv}(t)$ and obtain for the Volterra equation 3

$$m \frac{\mathrm{d}}{\mathrm{d}t} C^{vv}(t) = -C^{\nabla U v}(t) - C^{vv}(t) M_0 - \sum_{n=1}^{\infty} \frac{(-1)^n}{n!} \frac{\mathrm{d}^n C^{vv}(t)}{\mathrm{d}t^n} M_n. \tag{S4}$$

An open question is how to reliably estimate $\Gamma(t)$ from the moments M_n, which would be required for the GLE simulations used in this paper. As we have shown in the main text, the Volterra-approach fails when $\Delta t > \tau_k$, where the ansatz presented in[S2] might be a promising, yet challenging alternative to the GPO approach employed in this paper.

IV Fits of the Memory Kernels

We fit the memory functions for different discretizations Δt according to the five-component exponential fit in Eq. 5. Table S1 shows the resulting memory parameters $(\gamma_1, \tau_1, \ldots \gamma_5, \tau_5)$. The fits (colored) are compared to the memory kernels (gray) calculated using Eq. S1 in Fig. S2.

At discretizations of $\Delta t \geq 8\,\mathrm{ns}$, which is higher than the ones considered in this paper, the Volterra method experiences a complete breakdown, signified by the occurrence of negative values for $G(t > 0)$. As negative frictions are unphysical, we do not show the corresponding $G(t)$ in Fig. S2, nor in the main text.

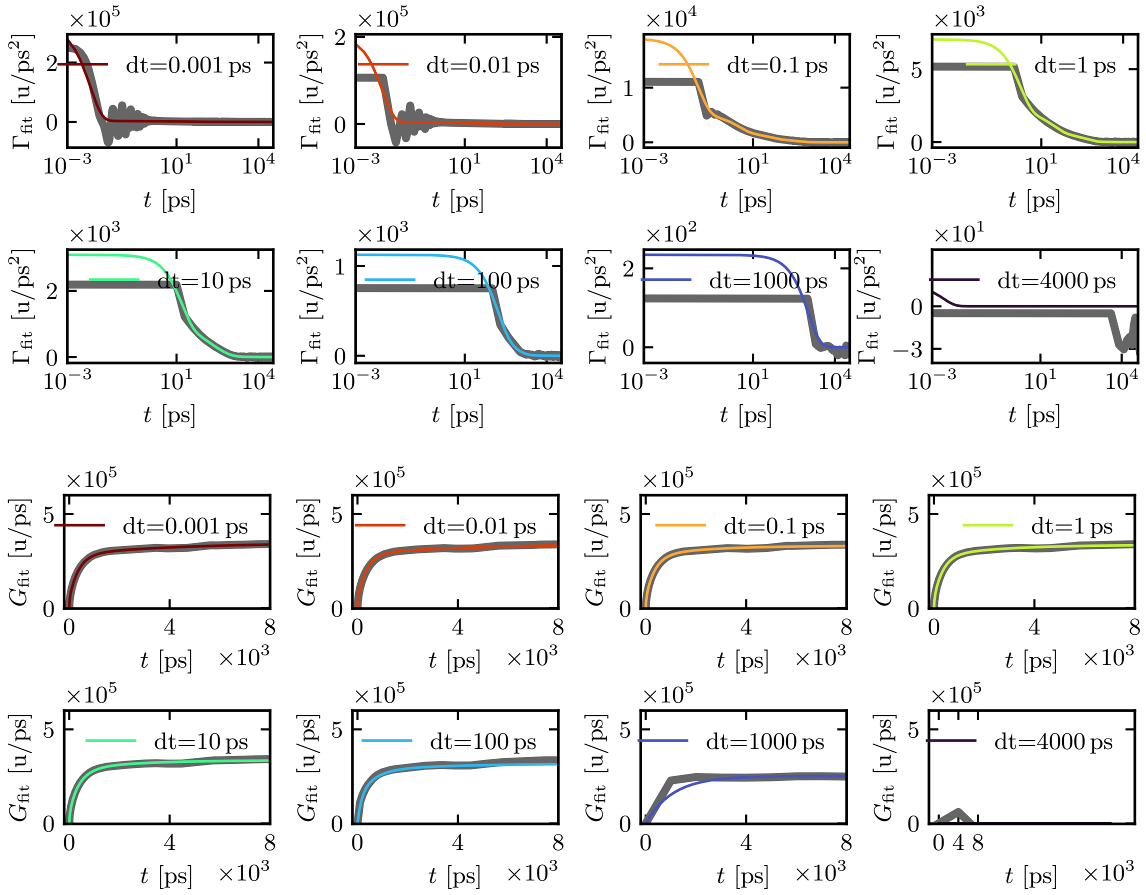

Figure S2: Memory extraction by the inversion of the Volterra equation 4 for different discretization times Δt, using MD data of Ala_9. We plot the fits of the memory kernels shown in Table S1 and their running integral as colored lines, the numerical data that is fitted is shown as gray lines.

Table S1: We show all memory times τ_i and the corresponding friction coefficients γ_i for the five-component exponential memory kernel fit according to Eq. 5 obtained from MD simulation data of Ala_9 subsampled at different discretization times Δt. The fits are plotted in Fig. S2. We also show the total friction $\gamma_{\text{tot}} = \sum_{i=1}^{5} \gamma_i$. The weights α_{mem} are chosen such that the mean-squared loss of the memory kernel and its running integral are approximately of the same order. Values are shown in units of ps for τ_i and Δt, u/ps for γ_i and atomic mass units u for m.

Δt	0.001	0.01	0.1	1.0	10.0	100.0	1000.0	4000.0
τ_1	6.8e−03	9.6e−03	9.9e−02	1.7e+00	1.5e+01	1.6e+02	1.1e+03	2.5e−03
γ_1	2.2e+03	1.9e+03	1.4e+03	8.5e+03	3.3e+04	1.4e+05	2.5e+05	3.9e−02
τ_2	1.8e+01	1.3e+01	2.8e+00	2.8e+01	1.0e+02	6.3e+02	5.4e+05	8.4e+05
γ_2	4.4e+04	3.6e+04	9.0e+03	4.0e+04	3.9e+04	1.5e+05	1.0e−04	5.0e−01
τ_3	3.7e+02	3.5e+02	3.2e+01	3.6e+02	4.3e+02	3.7e+03	5.7e+05	9.9e+05
γ_3	2.4e+05	2.5e+05	4.0e+04	2.3e+05	2.1e+05	3.6e+04	1.0e−04	2.2e−01
τ_4	4.1e+03	5.2e+03	3.6e+02	2.6e+03	1.8e+03	1.9e+04	6.2e+05	1.0e+06
γ_4	6.0e+04	5.6e+04	2.3e+05	4.6e+04	1.1e+02	0.0e+00	3.1e+04	1.3e+02
τ_5	5.7e+03	8.8e+04	2.3e+03	2.8e+03	2.4e+03	4.6e+04	7.8e+05	1.0e+06
γ_5	4.5e+03	2.8e+03	5.5e+04	1.4e+04	5.1e+04	7.0e+02	2.5e+03	3.6e+03
γ_{tot}	3.5e+05	3.5e+05	3.3e+05	3.4e+05	3.4e+05	3.2e+05	2.8e+05	3.7e+03
α_{mem}	1.0e−01	5.0e−03	1.0e−04	1.0e−05	5.0e−06	1.0e−09	1.0e−11	1.0e−14
m	3.1e+01	3.3e+01	1.0e+02	2.7e+03	1.2e+05	5.2e+06	1.6e+08	9.6e+08

V Markovian Embedding

The generalized Langevin equation

$$m\dot{v}(t) = -\nabla U[x(t)] - \int_{t_0}^{t} \mathrm{d}s\, \Gamma(t-s) v(t) + F_R(t), \tag{S5}$$

can be simulated by the following set of coupled Langevin equations

$$m\dot{v}(t) = -\nabla U[x(t)] + \sum_{i=1}^{5} \frac{\gamma_i}{\tau_i} \left(y_i(t) - x(t) \right), \tag{S6}$$

$$y_i(t) = -\frac{1}{\tau_i} \left(y_i(t) - x(t) \right) + \frac{\eta_i(t)}{\gamma_i}, \tag{S7}$$

where the random forces η_i fulfill the fluctuation-dissipation theorem $\langle \eta_i(t)\eta_j(t')\rangle = 2k_\mathrm{B}T\gamma_i\delta_{ij}\delta(t-t')$. The form of $\Gamma(t)$ is given by

$$\Gamma(t) = \sum_{i=1}^{5} \frac{\gamma_i}{\tau_i} \exp\left(-\frac{t}{\tau_i}\right). \tag{S8}$$

Solving for $y_i(t)$ in Eq. S7 and inserting into Eq. S6 yields the solution

$$\begin{aligned} m\dot{v}(t) = &-\nabla U[x(t)] - \int_{t_0}^{t} \mathrm{d}s \sum_{i=1}^{5} \frac{\gamma_i}{\tau_i} e^{-(t-s)/\tau_i} v(s) \\ &+ \int_{t_0}^{t} \mathrm{d}s \sum_{i=1}^{5} e^{-(t-s)/\tau_i} \frac{\eta_i(s)}{\tau_i} \\ &+ \sum_{i=1}^{5} \frac{\gamma_i}{\tau_i} e^{-(t-t_0)/\tau_i} \left(y_i(t_0) - x(t_0)\right), \end{aligned} \tag{S9}$$

which is identical to Eq. S5 with a random force which satisfies the fluctuation-dissipation theorem $\langle F_R(0)F_R(t)\rangle = k_\mathrm{B}T\Gamma(t)$. The mass m is computed via the equipartition theorem $m = k_\mathrm{B}T/\langle v^2\rangle$.

VI Breakdown of the Volterra Method

To investigate the failure of the Volterra method at discretizations higher than the memory time of $\tau_\mathrm{mem} = 1\,\mathrm{ns}$, we compare the velocity autocorrelation function $C^{vv}(t)$, the potential gradient-position correlation function $C^{\nabla Ux}(t)$ and the running integral over the memory kernel $G(t)$ for different discretization times (colored lines in Fig. S3). To show that the breakdown of the Volterra method is actually due to discretization effects of $C^{\nabla Ux}(t)$, we define two corrected discretized potential gradient-position correlation functions, which we construct by subsampling the full resolution correlation function $C^{\nabla Ux}_{\Delta t=11\,\mathrm{fs}}$ according to

$$\begin{aligned} \tilde{C}^{\nabla Ux}_{\Delta t=1\,\mathrm{ns}}[i] &= C^{\nabla Ux}_{\Delta t=1\,\mathrm{fs}}[10^6 i], \\ \tilde{C}^{\nabla Ux}_{\Delta t=4\,\mathrm{ns}}[i] &= C^{\nabla Ux}_{\Delta t=1\,\mathrm{fs}}[4\cdot 10^6 i], \end{aligned} \tag{S10}$$

where the striding factors 10^6 and $4 \cdot 10^6$ follow from the discretization ratios. Here, the subscript of $\tilde{C}^{\nabla Ux}_{\Delta t}$ refers to the discretization at which the correlation is computed. We depict the corrected discretized correlation functions $\tilde{C}^{\nabla Ux}_{\Delta t=}[i]$ as dashed lines in Figure S3B. By utilizing these corrected discretized correlations, we derive the memory kernels $G(t)$ shown as dashed lines in Fig S3C, which rather closely match the full-resolution kernel (red line, $\Delta t = 1\,\text{fs}$). This demonstrates that the breakdown of the Volterra method is caused by discretization effects of the $C^{\nabla Ux}(t)$ correlation function.

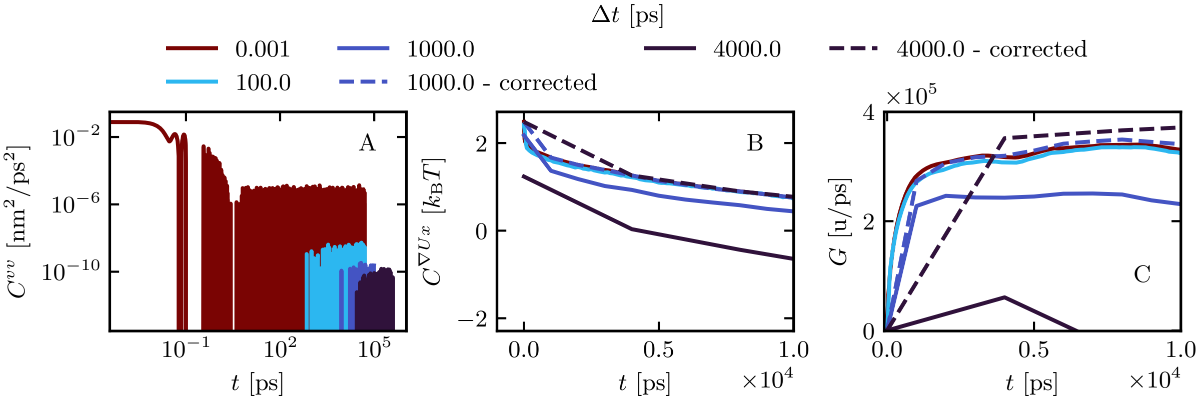

Figure S3: **A** Velocity autocorrelation $C^{vv}(t)$, **B** potential gradient-position correlation function $C^{\nabla Ux}(t)$, **C** running integral over the memory kernel $G(t)$. The colored solid lines show the data computed from discretized trajectories, the dashed lines in B show corrected discretized potential gradient-position correlation functions according to equation S10, the dashed lines in C show the corresponding results for $G(t)$.

VII Gaussian Process Optimization

Table S2: Bounds B for the individual parameters of $\theta = (\gamma_1, \tau_1, \ldots, \gamma_5, \tau_5)$ in the GPO.

	B_{τ_1}	B_{τ_2}	B_{τ_3}	B_{τ_4}	B_{τ_5}	B_{γ_1}	B_{γ_2}	B_{γ_3}	B_{γ_4}	B_{γ_5}
B_{lower}	0.9	5	10	30	30	10	10	10	10	10
B_{upper}	$1 \cdot 10^2$	$1 \cdot 10^3$	$1 \cdot 10^4$	$1 \cdot 10^4$	$1 \cdot 10^4$	$1 \cdot 10^4$	$2 \cdot 10^5$	$4 \cdot 10^5$	$6 \cdot 10^5$	$6 \cdot 10^5$

GPO is a widely known method, which will be briefly discussed here. For a more thorough explanation, the reader is referred to Ref.[S3] The quality of a set of GLE parameters $\theta =$

$(\gamma,\tau_1,\ldots,\gamma_5,\tau_5)$ over the parameter space given in Table S2 is characterized by the loss $\mathcal{L}$ in Eq. 6 via a GLE simulation. To enhance the efficiency of the GPO for a wide range of the input parameters θ, we use a logarithmic embedding $\tilde{\theta} = (\log_{10}\gamma_1, \log_{10}\tau_1, \ldots, \log_{10}\gamma_i, \log_{10}\tau_i)$. We assume that for a set of n simulation parameters $\tilde{\boldsymbol{\Theta}} = [\tilde{\theta}_1, \ldots, \tilde{\theta}_n]^T$ with results $\mathcal{L}(\tilde{\boldsymbol{\Theta}}) = [\mathcal{L}(\tilde{\theta}_1), \ldots, \mathcal{L}(\tilde{\theta}_n)]^T$, the loss follows a Gaussian distribution:

$$p\left(\begin{bmatrix}\mathcal{L}(\tilde{\theta}_1)\\ \ldots \\ \mathcal{L}(\tilde{\theta}_n)\end{bmatrix}\right) = \mathcal{N}\left(\begin{bmatrix}\mu(\tilde{\theta}_1)\\ \ldots \\ \mu(\tilde{\theta}_n)\end{bmatrix}, \begin{bmatrix}k(\tilde{\theta}_1,\tilde{\theta}_1) & \ldots & k(\tilde{\theta}_1,\tilde{\theta}_n)\\ \ldots & \ldots & \ldots \\ k(\tilde{\theta}_n,\tilde{\theta}_1) & \ldots & k(\tilde{\theta}_n,\tilde{\theta}_n)\end{bmatrix}\right), \tag{S11}$$

where $\mathcal{N}(\mu,\sigma)$ is a multivariate normal distribution with mean vector μ and covariance matrix σ. The process generating the set $\tilde{\boldsymbol{\Theta}}$ is a Gaussian process with the mean function $\mu(\tilde{\theta}_i)$ and the covariance function $k(\tilde{\theta}_i,\tilde{\theta}_j)$. The mean function is set to the scalar value $\mu(\tilde{\theta}_i) = \frac{1}{n}\sum_{i=1}^{n}\mathcal{L}(\tilde{\theta}_i)$. The log-likelihood follows as

$$\begin{aligned}\log p(\mathcal{L}(\tilde{\boldsymbol{\Theta}})) = -\frac{1}{2}(N\log 2\pi + \log\det\Big(k\left(\tilde{\boldsymbol{\Theta}},\tilde{\boldsymbol{\Theta}}\right)\Big)\\ + (\tilde{\boldsymbol{\Theta}}-\mu)^T k\left(\tilde{\boldsymbol{\Theta}},\tilde{\boldsymbol{\Theta}}\right)(\tilde{\boldsymbol{\Theta}}-\mu)),\end{aligned} \tag{S12}$$

where N is the dimension of $\tilde{\boldsymbol{\Theta}}$. To better model data containing noisy samples, a scalar noise parameter σ is added to the diagonal elements of the covariance matrix

$$\begin{aligned}\log p(\mathcal{L}(\tilde{\boldsymbol{\Theta}})) = -\frac{1}{2}(N\log 2\pi + \log\det\Big(k(\tilde{\boldsymbol{\Theta}},\tilde{\boldsymbol{\Theta}}) + \sigma^2 I\Big)\\ + (\tilde{\boldsymbol{\Theta}}-\mu)^T(k(\tilde{\boldsymbol{\Theta}},\tilde{\boldsymbol{\Theta}}) + \sigma^2 I)(\tilde{\boldsymbol{\Theta}}-\mu)),\end{aligned} \tag{S13}$$

where I is the identity matrix. The complexity of the loss function the process can represent is determined by the choice of $k(\tilde{\theta}_i,\tilde{\theta}_j)$. For $k(\tilde{\theta}_i,\tilde{\theta}_j)$, we choose the radial basis function (RBF) covariance function with an added constant c

$$k(\tilde{\theta}_i,\tilde{\theta}_j,s,l,c) = s^2\exp\left(-\frac{||\tilde{\theta}_i-\tilde{\theta}_j||^2}{2l^2}\right) + c, \tag{S14}$$

where $||\cdot||$ is the Euclidean distance. To determine the parameters s, l and c, the negative log-likelihood in Eq. S12 of the observed loss $\log \mathcal{L}(\tilde{\mathbf{\Theta}})$ is minimized, i.e. $\arg\min_{s,l,c} - \log p(\mathcal{L}(\tilde{\mathbf{\Theta}}))$. The noise level σ is kept constant at 0.005.

To predict the loss value at samples not observed so far, the data vector $\tilde{\mathbf{\Theta}}$ is split into the observed samples $\tilde{\mathbf{\Theta}}$ (where $\mathcal{L}(\tilde{\mathbf{\Theta}})$ is available) and unobserved samples $\tilde{\mathbf{\Theta}}_*$. The resulting covariance matrix has four blocks, the covariance of $\tilde{\mathbf{\Theta}}$ and $\tilde{\mathbf{\Theta}}_*$ with themselves and each other. The distribution

$$p\left(\begin{bmatrix} \mathcal{L}(\tilde{\mathbf{\Theta}}) \\ \mathcal{L}(\tilde{\mathbf{\Theta}}_*) \end{bmatrix}\right) = \mathcal{N}\left(\begin{bmatrix} \mu \\ \mu \end{bmatrix}, \begin{bmatrix} k(\tilde{\mathbf{\Theta}}, \tilde{\mathbf{\Theta}}) + \sigma^2 I & k(\tilde{\mathbf{\Theta}}, \tilde{\mathbf{\Theta}}_*) \\ k(\tilde{\mathbf{\Theta}}, \tilde{\mathbf{\Theta}}_*)^T & k(\tilde{\mathbf{\Theta}}_*, \tilde{\mathbf{\Theta}}_*) \end{bmatrix}\right), \tag{S15}$$

can be conditioned on the samples observed $\tilde{\mathbf{\Theta}}$ and their losses $\mathcal{L}(\tilde{\mathbf{\Theta}})$, yielding a normal distribution over $\mathcal{L}(\tilde{\mathbf{\Theta}}_*)$ with mean

$$M\left[\mathcal{L}(\tilde{\mathbf{\Theta}}_*)\right] = k(\tilde{\mathbf{\Theta}}, \tilde{\mathbf{\Theta}}_*)\left[k(\tilde{\mathbf{\Theta}}, \tilde{\mathbf{\Theta}}) + \sigma^2 I\right]^{-1} \mathcal{L}(\tilde{\mathbf{\Theta}}), \tag{S16}$$

and standard deviation

$$\begin{aligned} \Sigma\left[\mathcal{L}(\tilde{\mathbf{\Theta}}_*)\right] &= k(\tilde{\mathbf{\Theta}}_*, \tilde{\mathbf{\Theta}}_*) \\ &\quad - k(\tilde{\mathbf{\Theta}}, \tilde{\mathbf{\Theta}}_*)^T \left[k(\tilde{\mathbf{\Theta}}, \tilde{\mathbf{\Theta}}) + \sigma^2 I\right]^{-1} k(\tilde{\mathbf{\Theta}}, \tilde{\mathbf{\Theta}}_*). \end{aligned} \tag{S17}$$

To propose new samples, the loss space is explored or exploited. When exploiting, we maximize the expected improvement

$$\begin{aligned} EI(\tilde{\theta}_i) &= \mathbb{E}\left[\mathcal{L}_{\text{best}} + \xi - M[\mathcal{L}(\tilde{\theta}_i)]\right], \\ &= \left(\mathcal{L}_{\text{best}} + \xi - M[\mathcal{L}(\tilde{\theta}_i)]\right)\Phi\left(\frac{\mathcal{L}_{\text{best}} + \xi - M[\mathcal{L}(\tilde{\theta}_i)]}{\Sigma[\tilde{\theta}_i]}\right) \\ &\quad + \Sigma[\tilde{\theta}_i]\phi\left(\frac{\mathcal{L}_{\text{best}} + \xi - M[\mathcal{L}(\tilde{\theta}_i)]}{\Sigma[\tilde{\theta}_i]}\right), \end{aligned} \tag{S18}$$

where $\mathcal{L}_{\text{best}}$ is the best loss observed so far, ϕ is the probability density function and Φ

the cumulative distribution function of the standard normal distribution. To encourage sampling around the $\tilde{\theta}$ with the best loss, we smooth the expected improvement by adding the parameter $\xi = 0.05$.

When exploiting, we maximize the standard deviation of the Gaussian Process $\Sigma\left[\mathcal{L}(\tilde{\theta}_i)\right]$ in Eq. S17. In each GPO run, we begin with five samples drawn uniformly over the sample space given by the bounds in Table S2, then we explore for 25 iterations, followed by 270 iterations where we alternatingly explore and exploit.

Table S3 shows all results of the GP optimizations that are shown in Fig. 3 and 4 and discussed in the main text. Fig. S4 repeats the mean first-passage time calculations from Fig 3, while computing τ_{MFP} not using the discretization time Δt of the GP optimization but the full resolution of the GLE simulation, i.e. $\Delta t = 2\,\mathrm{fs}$

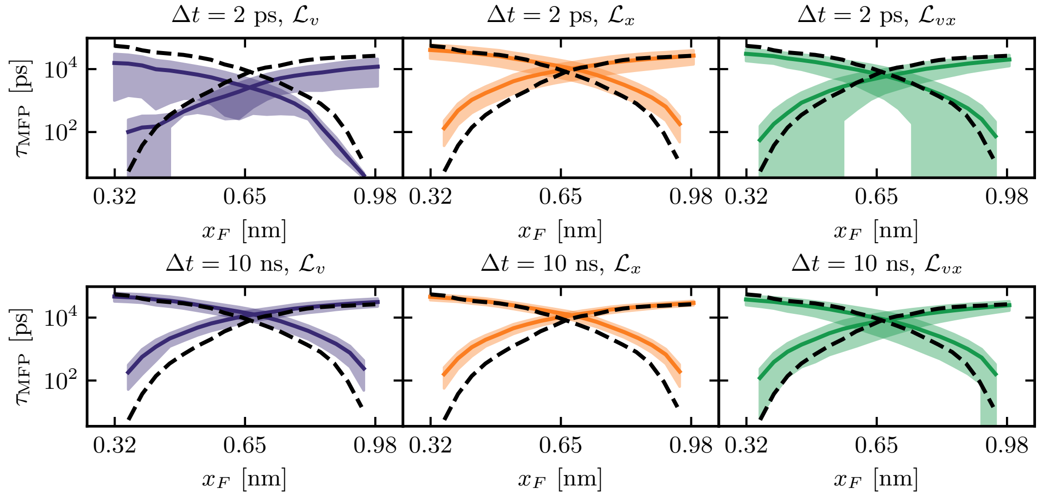

Figure S4: Comparison of τ_{MFP} starting from the folded state at $x_S = 0.32\,\mathrm{nm}$ and the unfolded state at $x_S = 0.98\,\mathrm{nm}$ as a function of the endpoint of τ_{MFP}, x_F, for GPO runs using $\mathcal{L}_v$, $\mathcal{L}_x$ and $\mathcal{L}_{vx}$ at $\Delta t = 2\,\mathrm{ps}$ and $\Delta t = 10\,\mathrm{ns}$. We show the mean of the ten best GPO runs (colored lines) and the standard deviation over the ten best runs (shaded area). Here, τ_{MFP} is computed from full-time resolution data, i.e. $\Delta t = 2\,\mathrm{fs}$. The MD reference is indicated by the black-broken line.

Table S3: GPO results from Fig 3 and Fig 4. We show the sum of the friction, $\gamma_{\mathrm{tot}} = \sum_{i=1}^{5} \gamma_i$, the mean first passage-time from $x = 0.98\,\mathrm{nm}$ to $x = 0.32\,\mathrm{nm}$, τ_{fold}, the mean first-passage time from $x = 0.32\,\mathrm{nm}$ to $x = 0.98\,\mathrm{nm}$, τ_{unfold}, and the weights α for $\mathcal{L}_{vx}$ with $\mathcal{L}_{vx} = \alpha\mathcal{L}_v + \mathcal{L}_x$. The folding and unfolding times are not comparable when computed from the data at different discretizations, so we compare τ_{MFP} computed from a GLE simulation trajectory at the full resolution of $\Delta t = 2\,\mathrm{fs}$. As in the main text, τ_{fold} and τ_{unfold} are computed via a mean over the ten best GPO runs. α is determined such that the magnitude of $\mathcal{L}_v$ and $\mathcal{L}_x$ is roughly equal. The table also shows the number of samples N_v and N_x used for the GPO losses, as given in Eq. 6.

$\mathcal{L}$	Δt[ps]	γ_{tot}[u/ps]	τ_{fold}[ps]	τ_{unfold}[ps]	α	N_v	N_x
$\mathcal{L}_v$	$2.0 \cdot 10^0$	$1.2 \cdot 10^5$	$9.8 \cdot 10^3$	$8.1 \cdot 10^3$	-	$1.0 \cdot 10^1$	-
$\mathcal{L}_x$	$2.0 \cdot 10^0$	$3.9 \cdot 10^5$	$4.6 \cdot 10^4$	$2.9 \cdot 10^4$	-	-	$2.5 \cdot 10^4$
$\mathcal{L}_{vx}$	$2.0 \cdot 10^0$	$3.9 \cdot 10^5$	$2.4 \cdot 10^4$	$1.6 \cdot 10^4$	$5.0 \cdot 10^3$	$1.0 \cdot 10^1$	$2.5 \cdot 10^4$
$\mathcal{L}_{vx}$	$2.0 \cdot 10^1$	$3.5 \cdot 10^5$	$3.6 \cdot 10^4$	$2.4 \cdot 10^4$	$1.0 \cdot 10^5$	$1.0 \cdot 10^1$	$2.5 \cdot 10^3$
$\mathcal{L}_{vx}$	$2.0 \cdot 10^2$	$3.7 \cdot 10^5$	$2.7 \cdot 10^4$	$1.9 \cdot 10^4$	$1.0 \cdot 10^5$	$1.0 \cdot 10^1$	$2.5 \cdot 10^2$
$\mathcal{L}_{vx}$	$2.0 \cdot 10^3$	$3.6 \cdot 10^5$	$3.7 \cdot 10^4$	$2.5 \cdot 10^4$	$1.0 \cdot 10^4$	$1.0 \cdot 10^1$	$2.5 \cdot 10^1$
$\mathcal{L}_{vx}$	$8.0 \cdot 10^3$	$3.8 \cdot 10^5$	$4.5 \cdot 10^4$	$2.8 \cdot 10^4$	$1.0 \cdot 10^5$	$4.0 \cdot 10^0$	$6.0 \cdot 10^0$
$\mathcal{L}_v$	$1.0 \cdot 10^4$	$3.5 \cdot 10^5$	$3.8 \cdot 10^4$	$3.0 \cdot 10^4$	-	$4.0 \cdot 10^0$	-
$\mathcal{L}_x$	$1.0 \cdot 10^4$	$3.6 \cdot 10^5$	$4.2 \cdot 10^4$	$2.8 \cdot 10^4$	-	-	$5.0 \cdot 10^0$
$\mathcal{L}_{vx}$	$1.0 \cdot 10^4$	$3.5 \cdot 10^5$	$4.2 \cdot 10^4$	$2.8 \cdot 10^4$	$1.0 \cdot 10^5$	$4.0 \cdot 10^0$	$5.0 \cdot 10^0$
$\mathcal{L}_{vx}$	$1.6 \cdot 10^4$	$3.8 \cdot 10^5$	$3.5 \cdot 10^4$	$2.2 \cdot 10^4$	$1.0 \cdot 10^5$	$4.0 \cdot 10^0$	$4.0 \cdot 10^0$
$\mathcal{L}_{vx}$	$2.5 \cdot 10^4$	$4.0 \cdot 10^5$	$3.7 \cdot 10^4$	$2.3 \cdot 10^4$	$1.0 \cdot 10^5$	$4.0 \cdot 10^0$	$4.0 \cdot 10^0$
$\mathcal{L}_{vx}$	$4.0 \cdot 10^4$	$3.6 \cdot 10^5$	$4.2 \cdot 10^4$	$2.7 \cdot 10^4$	$1.0 \cdot 10^5$	$4.0 \cdot 10^0$	$4.0 \cdot 10^0$
$\mathcal{L}_{vx}$	$8.0 \cdot 10^4$	$6.4 \cdot 10^5$	$6.7 \cdot 10^4$	$4.2 \cdot 10^4$	$1.0 \cdot 10^5$	$4.0 \cdot 10^0$	$4.0 \cdot 10^0$
$\mathcal{L}_{vx}$	$1.6 \cdot 10^5$	$2.2 \cdot 10^5$	$2.7 \cdot 10^4$	$1.8 \cdot 10^4$	$1.0 \cdot 10^5$	$4.0 \cdot 10^0$	$4.0 \cdot 10^0$
$\mathcal{L}_{vx}$	$2.4 \cdot 10^5$	$1.9 \cdot 10^5$	$2.0 \cdot 10^4$	$1.3 \cdot 10^4$	$1.0 \cdot 10^5$	$4.0 \cdot 10^0$	$4.0 \cdot 10^0$

VIII Interpolation of the Potential

To compute the correlation between the gradient of the potential and the position $C^{\nabla Ux}(t)$, we first estimate the probability $\rho(x)$ via a histogram using n_{bins}. Next, we compute the potential $U(x) = -k_{\mathrm{B}}T \ln \rho(x)$. To compute a trajectory $\nabla U[x(t)]$, we compute a numerical gradient of $U(x)$ and interpolate it such that it can be evaluated at every frame $x(t)$. Finally, we correlate the trajectories $\nabla U[x(t)]$ and $x(t)$, yielding $C^{\nabla Ux}(t)$. Here, we compare how $C^{\nabla Ux}(t)$ and $G(t)$ are influenced by the choice of n_{bins}. We also compare a linear to a cubic spline interpolation. Fig. S5B and F show that for both interpolations when choosing a low n_{bins}, the mean of $\nabla U[x(t)]$, $\langle \nabla U \rangle$, is far from zero. This is in contradiction to the fact that the simulation is stationary. With $\langle \nabla U \rangle$ far from zero, both $C^{\nabla Ux}(t)$ and $G(t)$ depend on the value of n_{bins}. When n_{bins} is increased, $\langle \nabla U \rangle$ converges to zero, and both interpolations agree on the shape of $C^{\nabla Ux}(t)$ in Fig. S5A and E, $G(t)$ in Fig. S5D and H, and $\Gamma(t)$ in Fig. S5C and G, independent of n_{bins}. This demonstrates that a consistent interpolation is possible. For the main text and in Appendix section IX, we use a linear interpolation with $n_{\mathrm{bins}} = 600$.

IX Discretized Velocity

To compute the velocities $v(t)$ from the positions $x(t)$, we compare two different differentiation schemes. We define a central gradient of order n:

$$v_i^{(n)} = \frac{x_{i+n/2} - x_{i-n/2}}{n\Delta t}. \tag{S19}$$

Second, we define smoothed positions $\tilde{x}(t)$, where we smooth the data by taking the running average over two adjacent points:

$$\tilde{x}_i = \frac{x_{i+1/2} - x_{i-1/2}}{2}. \tag{S20}$$

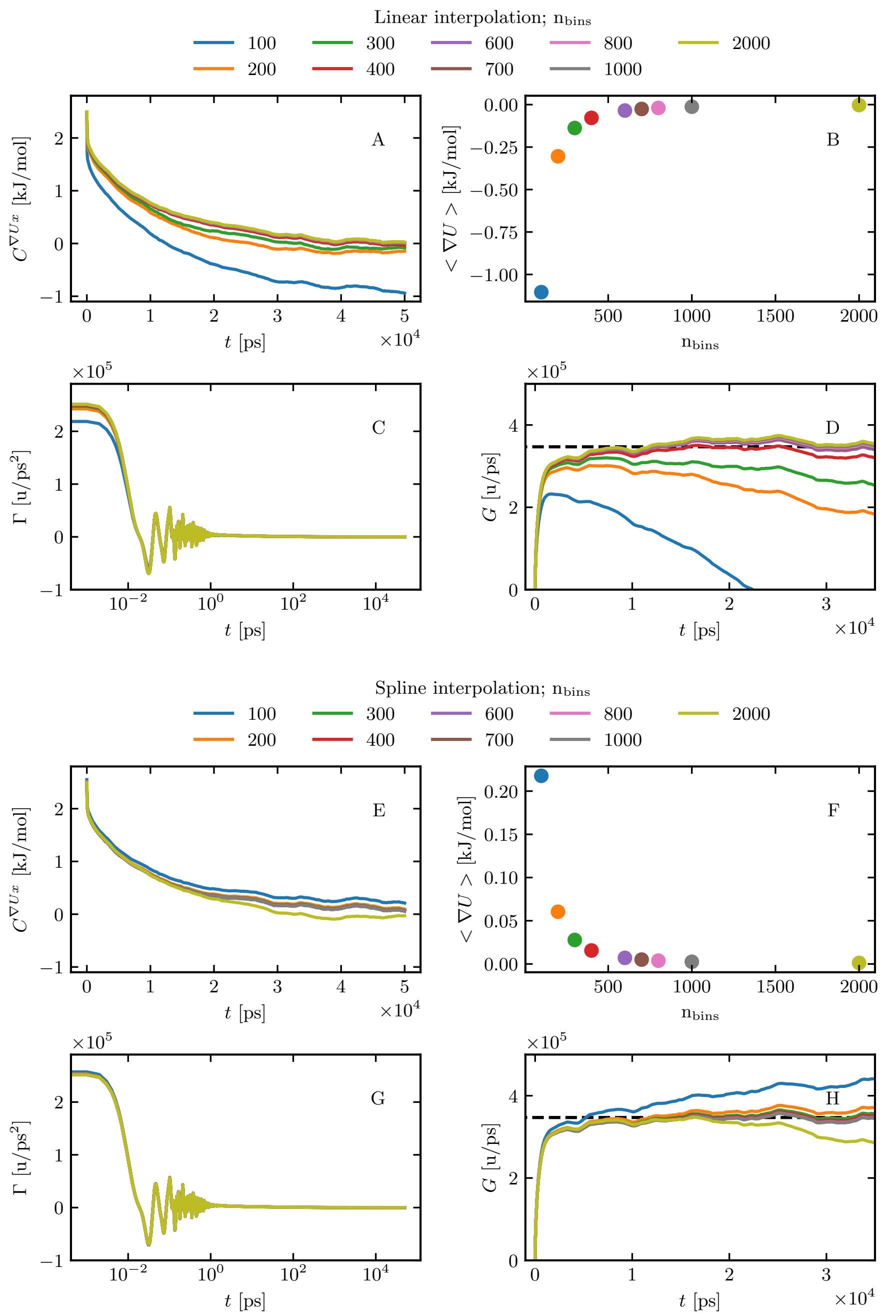

Figure S5: Memory extraction by the inversion of the Volterra equation 4 using MD data of Ala_9. We compare how the correlation between the gradient of the potential and the position $C^{\nabla U x}(t)$, the kernel $\Gamma(t)$ and the running integral of the kernel $G(t)$ depend on the number of interpolation points n_{bins} and interpolation type of the potential. The dependence on n_{bins} vanishes when $\langle \nabla U \rangle$ is close to zero. We show $C^{\nabla U x}(t)$ (**A**), $\langle \nabla U \rangle$ (**B**), $\Gamma(t)$ (**C**) and $G(t)$ (**D**) for different n_{bins} using a linear interpolation. We show the same for a cubic spline interpolation, i.e. $C^{\nabla U x}(t)$ (**E**), $\langle \nabla U \rangle$ (**F**), $\Gamma(t)$ (**G**) and $G(t)$ (**H**). In **D** and **H**, the black, dashed line indicates $\gamma_{\text{tot}} = \sum_i \gamma_i$ from Table 1.

We define smoothed positions of order m, where we apply the smoothing m times

$$\tilde{x}_i^{(m)} = \begin{cases} \tilde{x}_i & \text{if } m = 0 \\ (\tilde{x}_{i+1/2}^{(m-1)} + \tilde{x}_{i-1/2}^{(m-1)})/2 & \text{if } m \geq 1 \end{cases}. \tag{S21}$$

The smoothed gradient of order m is defined by smoothing the position m times and then applying the central gradient of order 1:

$$\tilde{v}_i^{(m)} = \frac{\tilde{x}_{i+1/2}^{(m)} - \tilde{x}_{i-1/2}^{(m)}}{2}. \tag{S22}$$

We note that the smoothed velocities of first-order equal the central gradient of second-order

$$\begin{aligned} \tilde{v}_i^{(m=1)} &= \frac{\tilde{x}_{i+1/2}^{(m=1)} - \tilde{x}_{i-1/2}^{(m=1)}}{\Delta t}, \\ &= \frac{(x_{i+1} + x_i)/2 - (x_i + x_{i-1})/2}{\Delta t}, \\ &= \frac{x_{i+1} - x_{i-1}}{2\Delta t}, \\ &= v_i^{(n=2)}. \end{aligned} \tag{S23}$$

Figures S6 and S7 show that the integrals over the kernel $G(t)$ that oscillate the least are obtained by the central differences of first order $v_i^{(n=1)}$. Accordingly, we use $v_i^{(n=1)}$ in the main text. In all cases, we compute the kernel $\Gamma(t)$ from $G(t)$ via a numerical gradient using central differences of second order, which alleviates the oscillations at low Δt.

References

(S1) Ayaz, C.; Tepper, L.; Brünig, F. N.; Kappler, J.; Daldrop, J. O.; Netz, R. R. Non-Markovian Modeling of Protein Folding. *Proc. Natl. Acad. Sci.* **2021**, *118*.

(S2) Cao, S.; Qiu, Y.; Kalin, M. L.; Huang, X. Integrative Generalized Master Equation: A

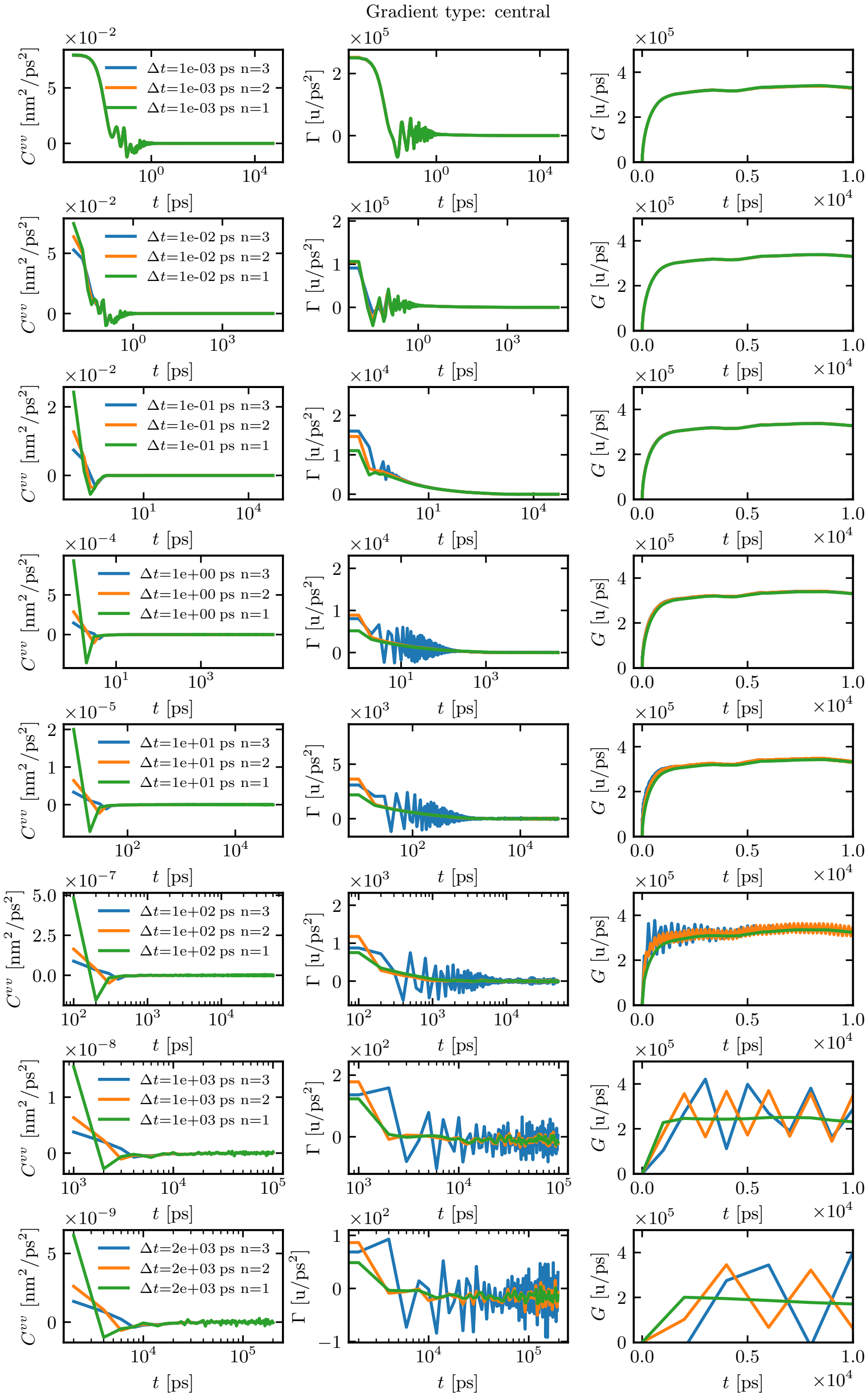

Figure S6: Memory extraction by the inversion of the Volterra equation 4 for different discretization times Δt, using MD data of Ala_9. Comparison of the memory kernels for central differences (Equation S19) of orders $n = 1$ to $n = 3$. The left column shows the velocity autocorrelation function $C^{vv}(t)$. The centre column shows the kernel $\Gamma(t)$. The right column shows the running integral over the kernel $G(t)$.

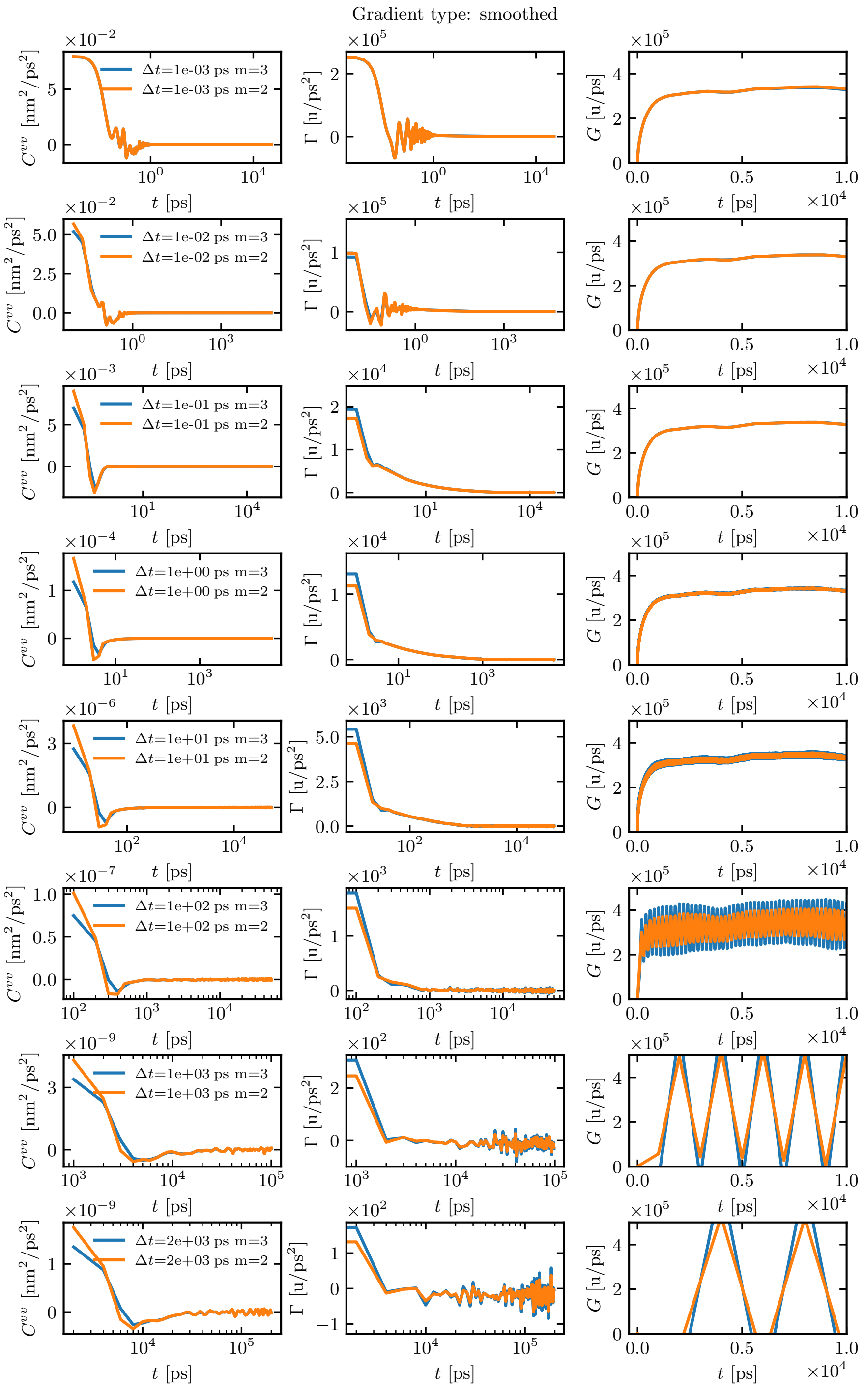

Figure S7: Memory extraction by the inversion of the Volterra equation 4 for different discretization times Δt, using MD data of Ala_9. Comparison of the memory kernels for smoothed differences (Equation S22) of orders $m = 2, 3$. The left column shows the velocity autocorrelation function $C^{vv}(t)$. The centre column shows the kernel $\Gamma(t)$. The right column shows the running integral over the kernel $G(t)$. We do not show order $m = 1$, as it is equivalent to the central gradient of order $n = 2$ shown in figure Fig. S6. (see Eq. S23).

Method to Study Long-Timescale Biomolecular Dynamics via the Integrals of Memory Kernels. *J. Chem. Phys.* **2023**, *159*, 134106.

(S3) Williams, Christopher K. I.; Rasmussen, Carl Edward *Gaussian Processes for Machine Learning*; MIT Press, 2006.
